# Pharmacologic rhythm control for atrial fibrillation with dronedarone versus sotalol in patients with and without heart failure

**DOI:** 10.1016/j.cpcardiol.2026.103332

**Published:** 2026-03-20

**Authors:** Silvio Nunes Augusto, David C. Kaelber, Mohamed Kanj, Shady Nakhla, W.H. Wilson Tang

**Affiliations:** a Department of Cardiovascular and Metabolic Sciences, Lerner Research Institute, Cleveland Clinic, Cleveland OH, USA; b The Center for Clinical Informatics Research and Education, The MetroHealth System, Cleveland OH, USA; c The Departments of Internal Medicine, Pediatrics, and Population and Quantitative Health Sciences, Case Western Reserve University, Cleveland OH, USA; d Department of Cardiovascular Medicine, Heart, Vascular and Thoracic Institute, Cleveland Clinic, Cleveland OH, USA; e Cleveland Clinic Lerner College of Medicine of Case Western Reserve University, Cleveland OH, USA; f Centennial Heart Cardiovascular Consultants, Nashville TN, USA

**Keywords:** Atrial fibrillation, Dronederone, Sotalol, Heart failure

Dronedarone and sotalol are Class III antiarrhythmic agents (AADs) used for rhythm control in patients with atrial fibrillation (AF), though both have limitations in patients with heart failure (HF). Sotalol carries class II beta-blocking activity and is generally avoided in patients with heart failure (HF) with reduced left ventricular ejection fraction (LVEF), in part due to risks of QT interval prolongation, bradycardia, fatigue, and hypotension. Dronedarone, on the other hand, is contraindicated in permanent AF and severe HF with left ventricular systolic dysfunction due to its association with increased cardiovascular mortality in these settings. Even though these findings were not consistently observed, dronedarone use is currently contraindicated in those with severe HF and left ventricular systolic dysfunction due to its association with increased early mortality due to worsening HF versus placebo.^1–3^ Recent analyses on this topic using several observational databases observed a significantly lower risk of cardiovascular hospitalization and ventricular arrhythmias, favoring dronedarone with the potential to reduce all-cause mortality.^4,5^

We conducted a retrospective cohort study using the TriNetX platform to compare adult patients across the United States (111 online at time of analysis; 157,870,495 patients on the network), aged 18 years or older, with atrial fibrillation (ICD-10-CM: I48) who were prescribed either dronedarone (RXNORM:233698) or sotalol (RXNORM:9947) for the first time between January 1st, 2010, and December 31st, 2021. The index date was defined as the date of the first prescription of the study drug. To ensure a new-user design,^1^ we excluded patients with prior diagnosis of AF before January 1, 2010, ensuring that the study AF diagnosis was incident within the modern coding era^2^; the first prescription of the study drug occurring at least one day after an AF diagnosis, establishing a clinically plausible drug-prescription sequence; and^3^ no exposure to the other drugs or other class III/IV AADs (ibutilide, dofetilide, bretylium, amiodarone) at any time, ensuring mutual exclusivity of exposure groups. Patients who were prescribed ibutilide (RXNORM: 41289), dofetilide (RXNORM: 49247), bretylium (RXNORM: 19685), or amiodarone (RXNORM: 703) were removed from this study. Propensity score matching (PSM) was used to balance demographics, comorbidities, prescriptions, and laboratory values, all with standardized mean differences <0.2, using the most recent value. Patients were stratified into two subcohorts: those with a diagnosis of HF (ICD-10-CM: I50) on or after January 1, 2010 (With HF, *n* = 4,904 matched pairs), and those with no HF diagnosis at any time (Without HF, *n* = 5,027 matched pairs). An additional combined cohort of all patients regardless of HF status was also analyzed (*n* = 8,765 matched pairs). Outcomes included all-cause mortality; heart failure (ICD-10 I50); acute HF, including acute systolic (ICD-10 I50.21), acute on chronic systolic (ICD-10 I50.23), acute diastolic (ICD-10 I50.31), acute on chronic diastolic (ICD-10 I50.33), acute combined systolic and diastolic (ICD-10 I50.41), acute on chronic combined systolic and diastolic (ICD-10 I50.43), acute right HF (ICD-10 I50.811), and acute on chronic right HF (ICD-10 I50.813); stroke, including cerebral infarction (ICD-10 I63), acute cerebrovascular insufficiency (ICD-10 I67.81), and cerebral atherosclerosis (ICD-10 I67.2); arrhythmia, including paroxysmal tachycardia (ICD-10 I47) and other cardiac arrhythmias (ICD-10 I49), major adverse cardiovascular events (MACE) that composed of acute myocardial infarction (ICD-10 I21), nontraumatic intracerebral hemorrhage (ICD-10 I61), cerebral infarction (ICD-10 I63), and sequelae of cerebrovascular disease (ICD-10 I69). Patients with a recorded history of each outcome prior to cohort entry were excluded from that outcome’s analysis. For all-cause mortality, time-to-event analysis was performed using Kaplan-Meier methods with log-rank testing and Cox proportional hazards modeling expressed as hazard ratios (HR) with 95% confidence intervals. For all other outcomes, results are shown as odds ratios (OR) with 95% confidence intervals. This retrospective study of deidentified data is exempt from informed consent by Institutional Review Board.

In the entire cohort, dronedarone prescription was associated with a lower risk for all outcomes tested (all-cause mortality: Hazard Ratio [HR] 0.88, 95% Confidence Interval [95%CI] 0.81, 0.95; HF: Odds Ratio [OR] 0.77, 95%CI 0.72, 0.82; MACE: OR 0.88, 95% CI 0.81, 0.95; Acute HF: OR 0.80, 95% CI 0.73, 0.88; Stroke: OR 0.89, 95% CI 0.8, 0.99; Arrhythmia OR 0.73, 95% CI 0.69, 0.78). We further investigated two sub-cohorts, one with HF and another without HF. We observed a lower risk of arrhythmia when dronedarone was prescribed compared to sotalol in patients without HF, and dronedarone prescription was associated with a significantly lower risk of all-cause mortality, MACE, and stroke compared to sotalol prescription (Figure. 1). In patients with HF, all-cause mortality, MACE, and stroke trended in favor of dronedarone prescription but did not reach statistical significance. Nevertheless, in the heart failure cohort, we observed that dronedarone prescription was associated with a lower risk of acute HF exacerbation compared to sotalol prescription in patients with HF (Fig. 1).

Acknowledging specific limitations from this analysis is warranted. First, because of how TriNetX limits cohort comparison to two cohorts per analysis, the absence of a more widely used prescription medication limits the broader clinical applicability of these findings. Second, AF subtype (paroxysmal, persistent, permanent) cannot be reliably ascertained from ICD-10-CM codes within the platform, and because misclassification of AF subtype likely exists in our cohort, this could meaningfully introduce bias into the estimates. Another important source of bias is the lack of LVEF data before and after the PSM (After PSM: All-comers: 11%; With HF: 13%; Without HF: 9.5%), which is an important parameter for HF phenotype characterization. Fourth, the TriNetX platform does not properly capture medication duration, adherence, dose adjustments, or whether patients switched treatments along the course of the prescription. Creating complex logic when querying cohorts decreases the sample size, as patients may have been treated for highly variable durations, limiting causal inference. The outcome relies on ICD-10-CM coding, which introduces risk of misclassification, particularly for HF hospitalizations (which may reflect historical rather than incident diagnoses) and stroke (which may capture prevalent rather than incident events, as prior stroke diagnoses cannot be fully excluded from administrative data), and arrythmia (which may involve variability in ECG morphology, and patient diversity). While the cohort design required the drug prescription to follow an AF diagnosis by at least one day, this does not constitute a formal washout period, and unmeasured prior exposures outside the TriNetX network cannot be excluded. Ultimately, given that residual confounding may be responsible for the observed differences, the results in this letter should be interpreted carefully. Despite inherited limitations of observational studies with PSM and potential selection bias of those favoring dronedarone in potentially less sick patients due to the black box warning of dronedarone prescription, these findings provide further reassurance that dronedarone can be an effective therapy for carefully selected patients with AF when directly compared with sotalol, even in patients with HF in whom concerns for HF exacerbation exist.

## Figures and Tables

**Fig. 1.**
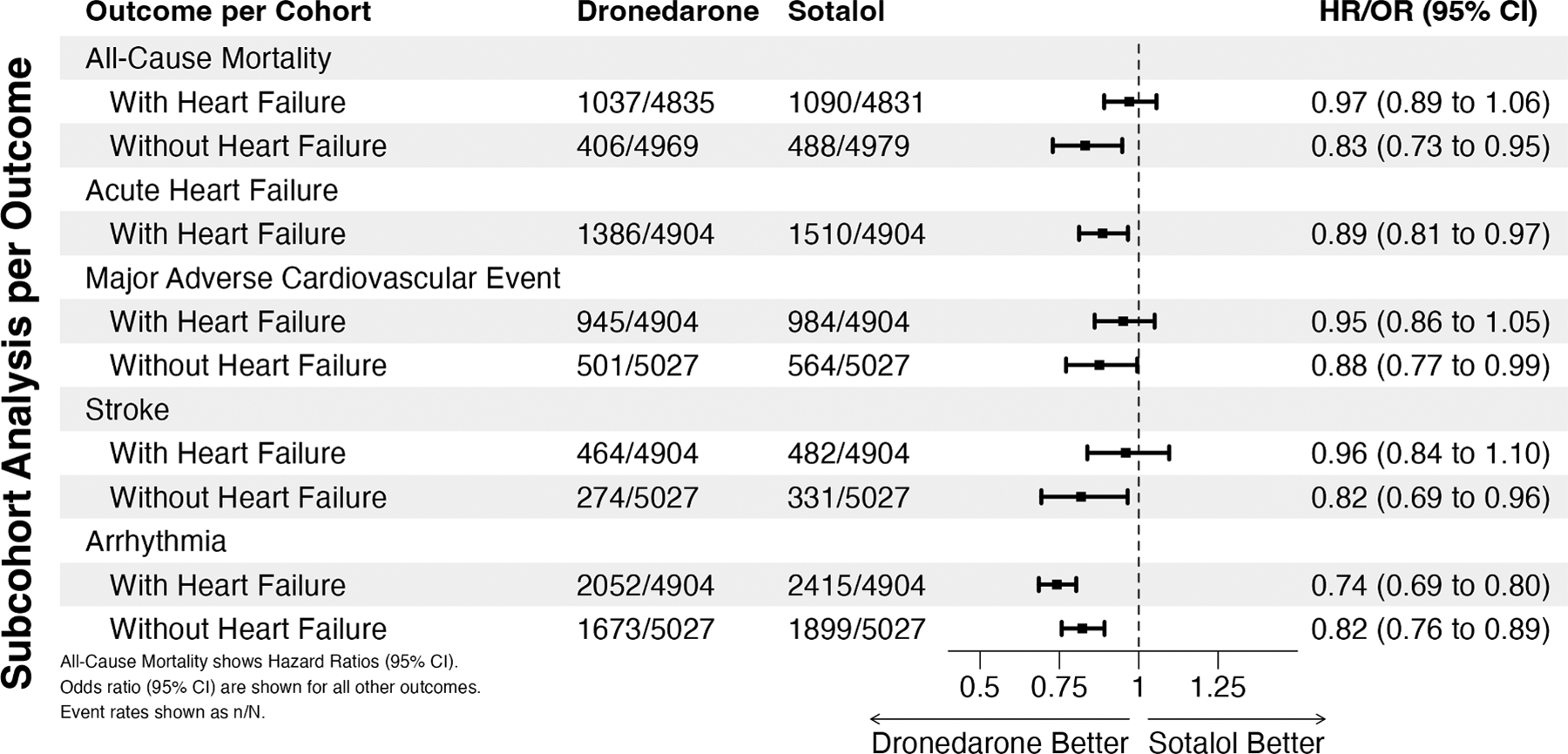
Forest plot showing propensity-score matched event rates and risks comparing dronedarone and sotalol prescriptions in patients with and without heart failure. All-cause mortality displays a hazard ratio (95% CI), whereas the other outcomes show an odds ratio (95% CI). Event rates are reported as events (n) per cohort (N).
